# Assemble-And-Match: A Novel Hybrid Tool for Enhancing Education and Research in Rational Structure Based Drug Design

**DOI:** 10.1038/s41598-017-18151-x

**Published:** 2018-01-16

**Authors:** Pouya Tavousi, Reza Amin, Sina Shahbazmohamadi

**Affiliations:** 1 Department of Pharmaceutical Sciences, University of Connecticut, Storrs, CT 06269 USA; 2 Department of Mechanical Engineering, University of Connecticut, Storrs, CT 06269 USA; 3 Department of Biomedical Engineering, University of Connecticut, Storrs, CT 06269 USA

## Abstract

Rational drug design is the process of finding new medication that can activate or inhibit the biofunction of a target molecule by binding to it and forming a molecular complex. Here, shape and charge complementarities between drug and target are key. To help find effective drug molecules out of a huge pool of possibilities, physical and computer aided tools have been developed. Former offers a tangible experience of the molecular interactions yet lacks measurement and evaluation capabilities. Latter enables accurate and fast evaluations, but does not deliver the interactive tangible experience of physical models. We introduce a novel hybrid model called “Assemble-And-Match” where, we enhance and combine the unique features of the two categories. Assemble-And-Match works based on fabrication of customized molecular fragments using our developed software and a 3D printer. Fragments are hinged to each other in different combinations and form flexible peptide chains, conformable to tertiary structures, to fit in the binding pocket of a (3D printed) target molecule. Through embedded measurement marks, the molecular model is reconstructed *in silico* and its properties are evaluated. We expect Assemble-And-Match tool can enable combination of visuospatial perception with *in silico* computational power to aid research and education in drug design.

## Introduction

Traditional drug discovery methods have a basis in trial-and-error^1^. In contrary, rational drug design approaches attempt to modulate a specific biological target in hope for a therapeutic value^2^. Typically, the ligand drug molecule must be designed such that it can attach to the binding site of the target strongly (i.e. with high affinity) and sometimes uniquely (i.e. with high specificity). This happens if there is a complimentary relationship between the drug and the target in shape (for affinity) and in charge (for specificity)^3^. Finding the best drug molecule candidate among a large library of possible ligands requires a way to evaluate the individual items^4,5^. Due to cost and time limitations, experimental screening of all possibilities is ruled out^6^. Therefore, several tools are employed to help narrow down the primary set of candidates. These tools that, are used both in research labs for conducting more efficient research studies and in the educational environments to raise the students’ comprehension of molecular structures and interactions, can be classified into two categories of physical^7,8^ and computer aided molecular models^9^.

Computational tools attempt to rank a set of either collected or enumerated candidates based on a scoring function^10^. The scoring function which is supposed to reflect the binding affinity between the drug and target molecules, is commonly derived semi-empirically^11^ but can also be obtained from density functional theory^12^ or ab-initio quantum chemistry techniques^13^. Scoring function can also be the result of knowledge based methods, where statistical and data mining tools are used for pattern extraction and data fitting from experimental binding affinity examples^14^. In a perfect world, the drug design screening tool will result in only one candidate solution. So, in turn, chemical synthesis of only one molecule will be sufficient. However in reality, because of the imperfection of the models, even after several iterations of computational evaluations, still several candidates must be chemically synthesized to compare their properties through experimental assays^15^.

The powerful features offered by computer aided drug design packages promise great advancements in the field. However, from the perspective of an expert pharmacist or immunologist who seeks a tangible and interactive experience of intermolecular interactions, computer tools may seem less handy in terms of the interfaces they provide (limited to computer-human communication only via keyboard, mouse and monitor^16^). Instead, the scientist would benefit much more from a model that, in addition to providing accurate computations and computer visualizations, also engages their visual and tactile sensory to enable them to more effectively apply their insight and experience in solving the drug design problem. In fact, although the human brain is usually slower than the computer to accomplish a certain number of mathematically well-defined tasks, but in many cases it is superior in solving complex tasks such as matching and assembling^17–20^. Virtual reality tools^21^ and haptic technology^22^ have been employed in an attempt to create environments that can provide a better sense of molecular complexes for drug designers. However, such technologies are still at their infancy and are often very costly. In a study conducted on the usage of tangible interfaces for structural molecular biology, reported in ref.^23^, the authors concluded that such interfaces are advantageous over solely computer visualization methods as they can produce a multisensory engagement, provide the capability of analog computation, offer a natural and intuitive mechanism for manipulation and exploration, and finally provide overview and detail simultaneously.

Although the advent of computer tools somewhat shifted the interest of scientific community from physical to the digital models, the unique insightful and interactive features of physical models keep them still in play. In fact, their popularity for qualitative studies and teaching the molecular concepts has been rising in the past few years^7,24^. The three main categories of physical molecular models are skeletal, ball-and-stick and space-filling. In the skeletal models, bonds and atoms are represented by rods and the intersections of them respectively. In the ball-and-stick model, bonds are rods and atoms are represented by spheres. The fact that atom radii here are significantly scaled down, makes this model incapable of representing surface and space-occupying properties of chemical molecules. The space-filling model represents atoms by spheres where the radius of sphere is proportional to the radius of atom and the distance between the centers of spheres is proportional to the distance between nuclei of atoms. Space-filling models are usually used to represent the interacting surface of the molecule with other molecules^25,26^. Space-filling models are also known as CPK models named after Robert Corey, Linus Pauling, and Walter Koltun who were pioneers in developing molecular models. Depending on its type, a molecular model may only represent the whole molecule as one piece or it may reflect the construction of the molecule from smaller constituents. The composite Nicholson model in which amino acid or nucleic acid residues act as the construction pieces for forming macromolecules^27^ is a good example of the latter.

Tracing the development of physical tools in the recent years, one will observe the wide spread usage of 3D printing technology in advancing more sophisticated models^28,29,30^. This has been so, mainly because additive manufacturing has in one way overcome some of the challenges on the way of fabricating complex geometries, and in another way has made custom design more affordable for almost everyone. The current 3D printing technologies and the impact of 3D printed models on students’ comprehension and retention of structural information are reviewed in ref.^31^. In one study reported in ref.^32^, the authors attempted to address the difficulty that students face in processing the macromolecular structures by creating four different 3D physical models followed by testing them on students in order to probe the effectiveness of these models on learning. The authors concluded that, with the use of such models, the barrier to understanding complex structures by students can be lowered.

One thing that pertains to all physical models is their role in enhancing the visuospatial ability which seems to be a critical factor in perceiving concepts of chemistry, biochemistry and the related topics. The longitudinal findings reported in ref.^33^ support the crucial role of spatial ability in developing expertise in STEM. Also^34^, reports on an examination conducted on the role of visuospatial cognition in chemistry learning, in which the authors suggest that an experience in manipulating concrete models is crucial in helping students solve chemistry problems. Furthermore, the study in ref.^35^ compares the learning of students that utilize physical models versus those that use textbooks and 3D computer models for learning imaging anatomy, showing a significant lead of the first group in terms of learning and comprehension. In another case, the controlled study on learning of students conducted in ref.^36^ suggested that combining hand-held models with computer imaging programs helps with answering questions on molecular structure and function.

Despite the wide usage of physical molecular models for qualitative representations, they were rarely used for rigorous quantitative evaluation of molecular systems, both in education and research, until very recently. Chakroberty *et al*. demonstrated in refs^16,37,38^ a successful attempt to build a flexible scaled physical model so called Peppytides which provides a coarse grained representation of the peptide chain for protein folding applications. The limited flexibility is manifested through rotations that are present only in the backbone. Further, the embedded magnets in their design simulate the short-range barriers on the backbone torsion angles as well as the long-range hydrogen bonds. A marker-less tracking method has been presented in ref.^39^ to recover the 3D structure of these models from a smart phone video. The self-assisting protein folding kit presented in ref.^40^ is another good example of flexible models for the polypeptide backbone. With the developed model, the authors produced an idealized model of the Triosephosphate Isomerase Mutase enzyme (TIM). The model is aimed at being used by students for a step-by-step investigation into the nature of protein folding.

A similar way of thinking in structure-based drug design applications and teaching of molecular interactions seems to be missing. Development of physical models that grant flexibility of the ligand, evoke tactile and visual sensory and allow accurate measurements can make a breakthrough in the advancement of drug design tools and further, provide the students with highly interactive tools that can call upon their cognitive and visuospatial abilities toward faster and more efficient learning of the concepts.

In this work, we will report the design and verification of a novel hybrid tool, for enhancing education and research in structure-based drug design, including physical and computational components. The tool which is called Assemble-And-Match enables custom fabrication of ligand molecular fragments and the receptor molecule using a computer framework and a desktop 3D printer. The user can then exploit their visual and tactile sensory to assemble the fragments in different combinations and alter the conformation of the peptide chain to reach at the best fit between the ligand and the receptor. They will then report the torsion angles through the embedded measurement marks to the software to reconstruct the model and evaluate various properties such as quality of the binding fit.

The Assemble-And-Match model provides a sophisticated and detailed representation that enables side chain rotations in addition to the main chain flexibilities. In addition, per selection of the user, side chains of the receptor can also be mobile. Further, the molecular fragments are not limited to those extracted from standard amino acids. Rather, the user can apply their insight to choose an arbitrary lumped group of atoms as a molecular fragment.

The complexity of assembling task is minimized by 3D printing the molecular fragments and the receptor as one piece components in such a way that no additional components are required for making connections between them (in contrast to the Peppytide design that uses a set of screw, nut and nylon spacer for constructing a joint and to the model presented in ref.^40^ where, chain’s alpha carbons must be strung successively using an elastic monofilament). The pieces are printed in such a way that guide the user in making the right connections, eliminating the possibility of any wrong assembly. This promotes fast plug and play which in turn enables rapid testing of several different combinations of construction fragments, leading to a handy and efficient tool for drug design applications. Also, the fact that collision between atoms is automatically avoided in the physical model (as a result of interaction between solid surfaces) results in: trimming a large portion of the configuration space, screening of which takes huge computational time in computer-based drug design; and modeling molecules as impenetrable concrete objects, helping students distinguish between favorable and unfavorable interactions.

The developed software is comprised of two modules, namely design and analysis. Using the design module, CAD models of the molecular fragments and the receptor can be generated in a highly customized fashion, enabling the drug designer to tailor the tool based on their needs. Easy connection between the physical and computer models is facilitated through embedded 3D printed measurement marks, size and accuracy of which are customizable. The analysis module then reconstructs the physical model *in silico* and uses it to evaluate various properties of the molecular system including different forms of energy.

**Figure 1:**
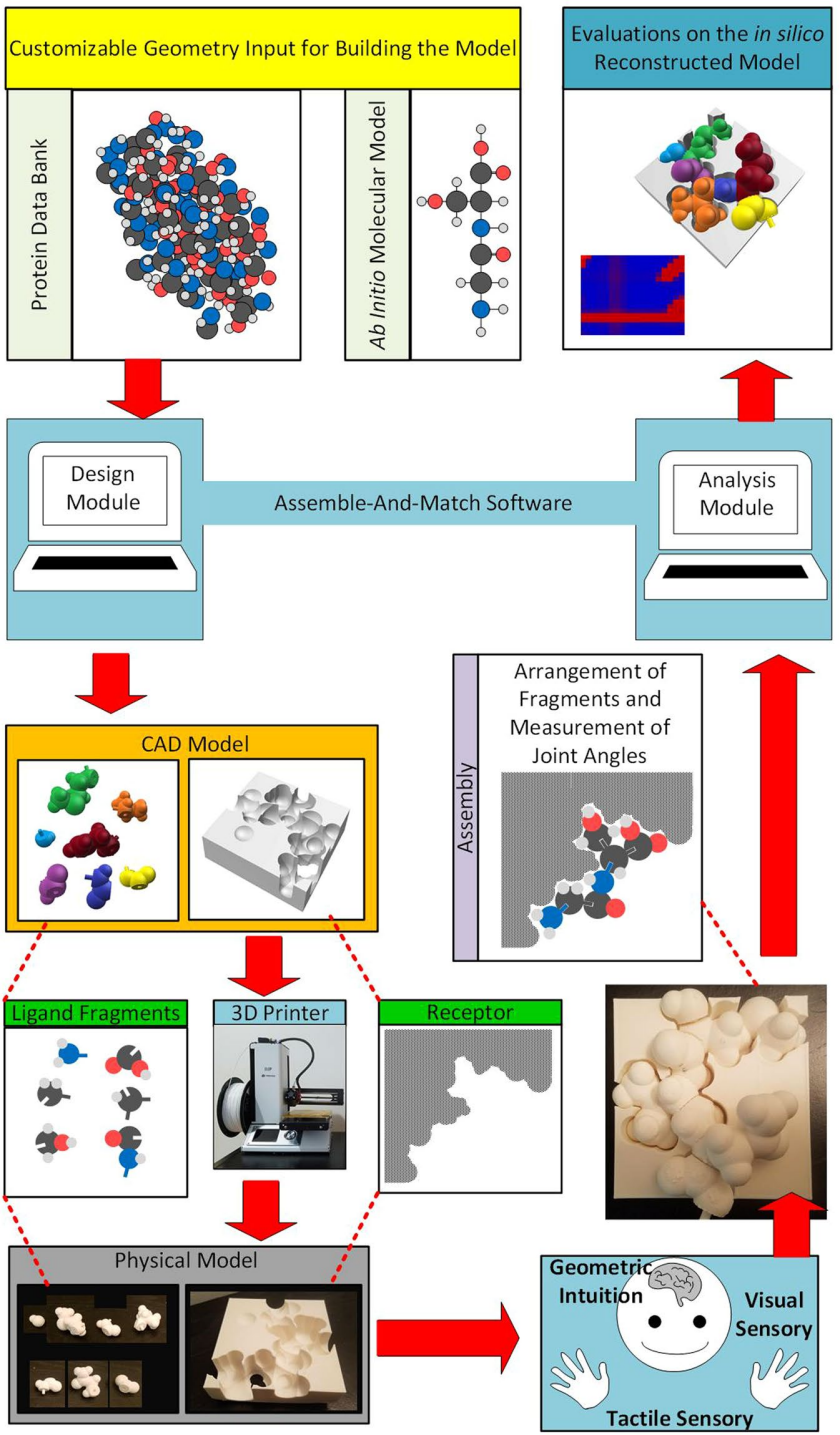
The Assemble-And-Match software extracts molecular structure information from protein data bank or uses a custom *ab initio* model to generate CAD models of the receptor and ligand fragments. These are fabricated using a 3D printer. The user then applies their geometric intuition and insight to form high affinity ligand-receptor complexes. Following that, they report the favorable conformations to the software by measuring the torsion angles. The software enables evaluation of various properties of the complex.

The simple design of the tool has made it easy to afford and use for almost any laboratory that works in the field, as well as any educational institute. Moreover, by exploiting the molding technology, low-cost molecular kits can be manufactured to be affordable by individual students. Figure 1 summarizes the functionality of Assemble-And-Match tool and the connection between its components.

## Material and Methods

### Molecular Model

**Figure 2:**
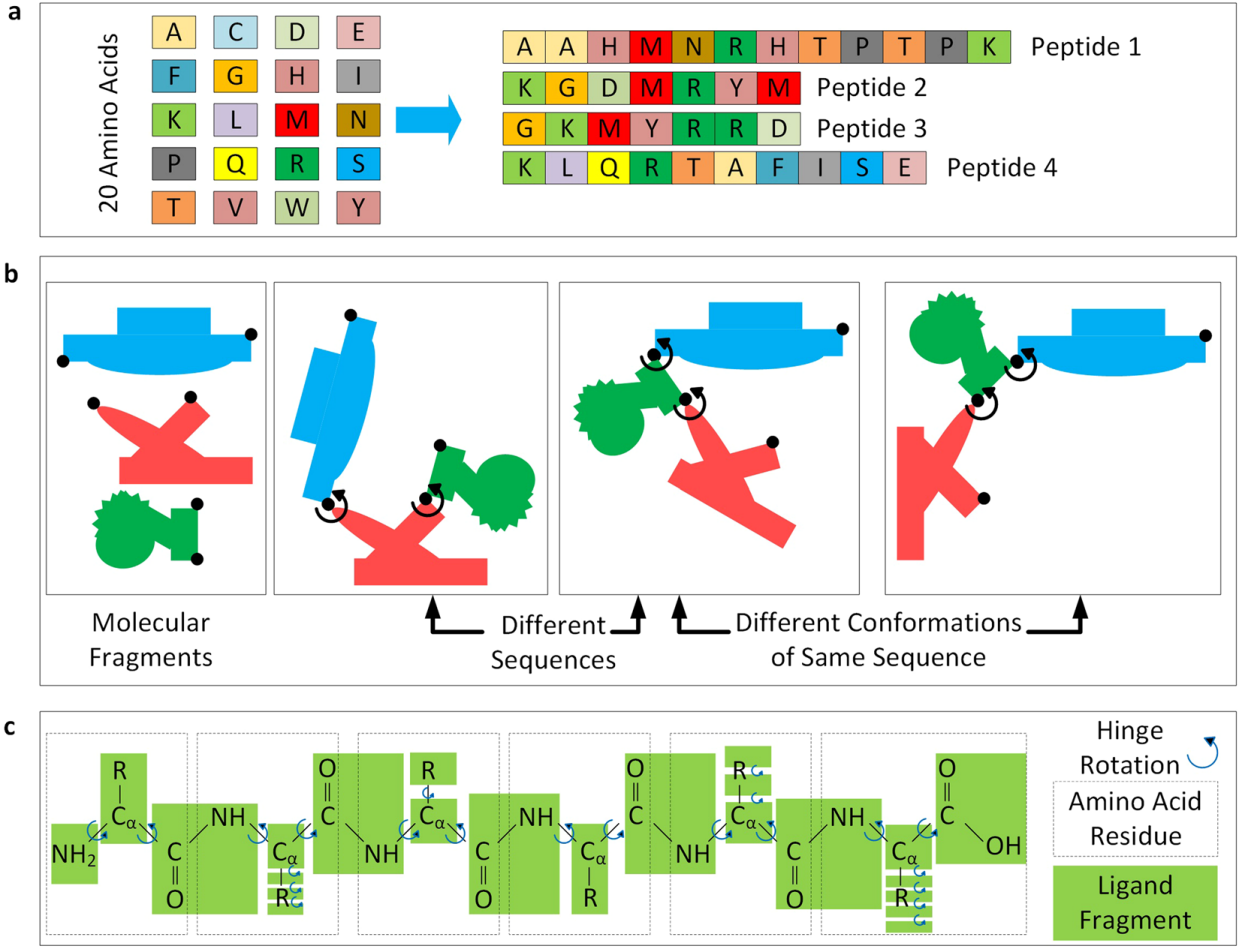
(**a**) Only 20 amino acids can form infinite number of different peptides. (**b**) Two things can completely determine the final structure of a peptide: the sequence of amino acids and how the torsion angles are set. (**c**) The ligand rigid fragments are distinct from the amino acid residues.

The Assemble-And-Match strategy for drug design is based upon the assumption that every drug molecule is made up of a finite number of building block components. One can think of these building blocks as simple LEGO pieces employed for constructing a more complex structure. Herein, we focus only on peptide-based drugs (This however, is potentially extendable to other types of drugs.). Peptides are polymeric chains formed by sequential linking of a number of building blocks so called amino acids. There are only 20 different types of amino acids naturally available. The diversity among different peptides rises from the different sequential arrangements of these amino acids, just like how only 26 letters make all the different English phrases (Fig. 2a).

Each peptide molecule consists of a set of atoms that are put together by means a number of covalent bonds along a chain. These covalent bonds have one thing in common: they restrain the motion of atoms in the molecule relative to each other. However, they do so to different extents. Some completely rigidify a part of the molecule. Some other, allow some flexibility: it is observed that rotation is allowed around certain single covalent bonds within the molecule. In fact, one can think of the peptide chain as number of rigid pieces hinged together by these single bonds. This chain can adopt different conformations as a result of the permitted rotations. These rotations are parameterized by introducing a set of torsion angles, one for each flex region in the main chain or any of the side chains^41^. Each amino acid contributes 3 main chain angles (*ϕ*, *ψ* and *ω*) and 0 to 4 side chain angles (*χ*_1_ to *χ*_4_) to this set. It is known that *ω* always gets locked at around 0° or 180°^41^. Therefore the conformation of a peptide chain can be completely described only with a set of *ϕ*, *ψ* and *χ*_*i*_ (1 ≤ *i* ≤ 4) values. As a result, two things can completely determine the final shape (i.e, 3D structure) of a peptide: the sequence of amino acids and how the torsion angles are set (Fig. 2b).

**Figure 3:**
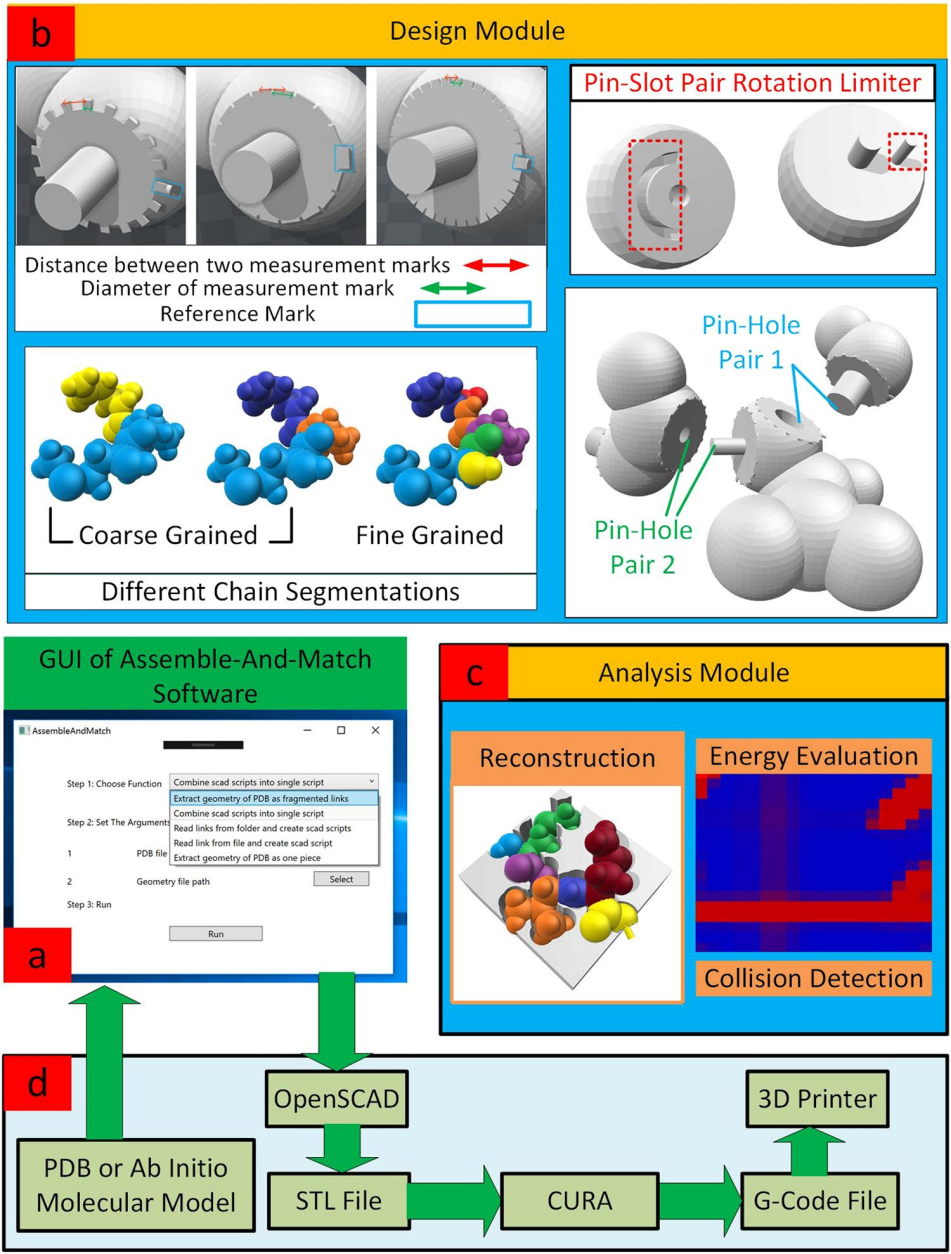
(**a**) A snapshot of Assemble-And-Match software user interface. (**b**) Highly customizable physical models promoted by the design module of the software. (**c**) *In silico* reconstruction and evaluation of molecular complexes enabled by the analysis module of the software. (**d**) Conjunction with external data, software and hardware for an effective customized design process.

Note that although amino acids are natural building blocks of the peptide chain, they do not reflect the rigid regions of the chain molecule. In fact the ligand rigid fragments are the result of a different segmentation of the chain (Fig. 2c). Each main chain fragment can be connected to one, two or three other pieces, one on the left (closer to the N-terminus of the chain), one on the right (closer to the C-terminus of the chain), and maybe one side chain fragment. Each side chain fragment can be connected to one or two other fragments. Herein, we call theses connection spots on each piece the male and female ports. The pieces are designed in such a way that, the intuitive mind can connect them to each other in the proper fashion. In fact, the revolute joints (responsible for the flexibility of the assembled molecule) are embedded in the model by means of male/female pairs, i.e., one protrusion on one piece is paired with a hole on another piece (Fig. 3b top right). For an assembly to reflect a real peptide chain, it is important that the male port of one rigid fragment is connected to only the female port of another rigid fragment and vice versa. Custom setting of shape and size of ports advocates differentiation between correct and incorrect connections and completely eliminates the possibility of any faulty assembly by the user (Fig. 3b bottom right). Measurement marks are embedded on the junction between the two pieces by which the user can read and report the associated torsion angles (Fig. 3b top left) to reconstruct the molecular system *in silico*.

### Printing Equipment

In general, any 3D printing equipment can be used to fabricate the construction pieces. For the examples shown in this paper, we have used Monoprice Select Mini 3D Printer (Monoprice, CA, USA) with heated plate along with 1.75 mm ABS filament (HATCHBOX, CA, USA). The follwing conditions were used for printing: temperature of the heating bed = 70 °C; temperature in nozzle = 230–240 °C; layer height = 0.2 mm; shell thickness = 0.8 mm; bottom/top thickness = 0.6 mm; fill density = 25%; print speed = 50 mm/s. Also, Kapton tape (TapesMaster, CA, USA) was adhered on top of the printing platform and to help the bottom layer of the print stick well to the bed, ABS was dissolved in acetone and rubbed using cotton to deposit a thin layer of ABS on the Kapton tape.

### Software

The software developed for the Assemble-And-Match drug design tool consists of two modules, namely design and analysis. The design module delivers a highly customizable arrangement for architecting ligand fragments as well as molecular receptors. The user has control on the location of flexible and rigid regions, meaning that their hands are open to either use the automatic segmentation, so called as “fine fragmentation”, described in the Molecular Model section and used in refs^41–44^, or rather apply their structural biochemistry insight to introduce an alternative segmentation, so called as “coarse fragmentation”. An example of the latter would be the instances where almost rigid molecular domains are present in the system (Fig. 3b bottom left). They can also specify the range of rotation around each joint (Fig. 3b top right). Further, the connection between fragments is customizable in terms of parameters such as joint diameter and depth (Fig. 3b bottom right). As mentioned earlier, this is advantageous for the physical interface by helping the user avoid faulty connections. Depending on the problem at hand and the actual size of macromolecules, the scale can be adjusted before printing. One can also alter the radii of atoms to attain various representations of the molecular system for different applications ranging from skeletal to solvent accessible surface area and beyond. Further, the size and shape of the measurement marks can be adjusted by the user (Fig. 3b top left).

The computational heart of the design module, in conjunction with external database, existing open source software packages and additive manufacturing technology empowers an effective design procedure. PDB files are fed to the software to extract rigid fragments based on user custom settings or alternatively molecular fragments are generated based on a custom *ab initio* model, input by the user. The software then tailors the CAD models of the receptor and the ligand fragments based on the settings chosen by the user. This is conducted by generating SCAD scripts and feeding them to OPENSCAD^45^ which is an open source CAD package that can render the generated pieces (i.e., ligand fragments and receptor) and export them as STL files^46^. The generated STL files are then converted through CURA free 3D printing software^47^ to G-Code files (needed for the specific 3D printer used in this work) that contain the 3D printing instructions needed by the 3D printer (Fig. 3d).

The analysis module of the software uses the reported measurement by the user to reconstruct the model *in silico* and evaluate the candidate structures discovered via the physical model (Fig. 3c). Although powerful (and in some cases unique) capabilities are offered through the built-in analysis module, the user may choose to employ any other state-of-the-art computational package for their analysis job. This communication is made possible through standard molecular file types such as PDB, mol2, etc. This way, not only none of the powerful features of the computational packages are lost, but also the user can enjoy the automatic and customizable design of CAD models from the molecular database or *ab initio* molecular models.

A user friendly graphical user interface (GUI) has been developed to make it easier for the drug designer to make most of the capabilities offered by the software.

### Physical Interface

Having the rigid receptor and the ligand fragments tailored through the software and fabricated by the 3D printer, the user, using their geometric intuition, can then select any combination of the ligand pieces, followed by putting them together to form a chain and then try to conform the chain aiming to arrive at a 3D structure that properly fills the binding pocket on the receptor. If they find a good conformation that matches the receptor, then they read the torsion angles via embedded measurement marks (we are planning to make improvements by adding a feature that the angles are read by a phone camera) and then report them along with the sequence to reconstruct the peptide structure in the software to evaluate the properties of the resulting complex. Otherwise, they will continue by trying another assemblage of the rigid fragments until they reach a good match. The insight that the user will attain during this process will help them make better choices in the following trials, both in terms of the selection of rigid fragments and their arrangement and setting of torsion angles.

### Data availability

The datasets analysed during the current study are available in the Protein Data Bank repository, https://www.rcsb.org/pdb/home/home.do.

## Results and Discussion

We devised a few test cases and demonstrations to investigate different aspects of the functionality of the developed Assemble-And-Match tool.

### Semi-Perfect Template

**Figure 4:**
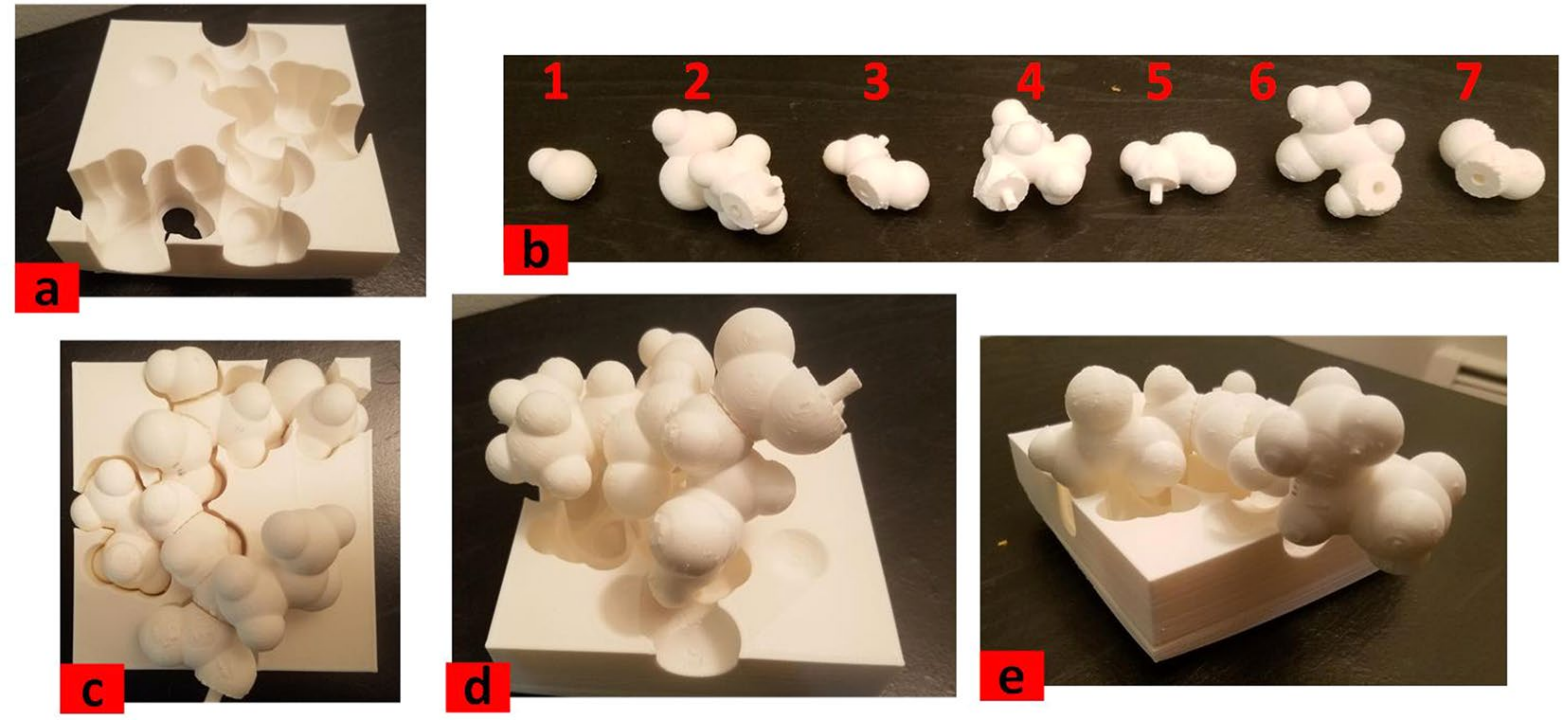
(**a**) The semi-perfect template acting as receptor (**b**) Ligand fragments (**c**) Perfect match acheived by proper selection of sequence and torsion angles (**d**) Imperfect match due to off-value torsion angles (**e**) Imperfect match due to scrambled sequence.

The goal of the Assemble-And-Match approach is to provide the drug designer with the proper tool so they can combine their visual and tactile sensory with their insight and experience to find good ligand/receptor matches. In fact, two factors contribute to the quality of the final outcome: efficiency of the tool and how well the tool is used by the user. Thus, it is worth having a test case in which the two factors are decomposed for a more objective evaluation of the tool. For this, we have taken a portion of a random peptide chain structure from PDB (3.5 amino acids starting from the N-terminus of a protein with PDB code of 2MZU) and did the following: (a) Fabricated a “semi-perfect” template for the structure by geometric subtraction of the spherical atoms and their upward sweeps from a rectangular box that was half the size of the bounding box of all atom centers. The resulting shape acted as the receptor (Fig. 4a); (b) Segmented the peptide chain to get 7 ligand fragments (Fig. 4b). For simplicity, the side chain torsion angles were assumed to be locked; (c) Assembled the peptide chain with different sequential combinations and different settings of torsion angles and attempted to match the resulting structures with the receptor. With the right sequential order of ligand pieces and torsion angles, the perfect match between the ligand chain and the receptor was obtained (Fig. 4c). Figure 4d depicts a case that the sequential order of ligand pieces is right but the torsion angles are off 0°, 60°, 40°, 40°, 80° and 40° respectively. Similarly, Fig. 4e shows a case that the order of fragments is scrambled namely, 1 → 2 → 5 → 6 → 7 → 3 → 4 instead of 1 → 2 → 3 → 4 → 5 → 6 → 7. Notice that for both of these cases, a perfect match could not be achieved.

### Generating Ramachandran Plot

**Figure 5:**
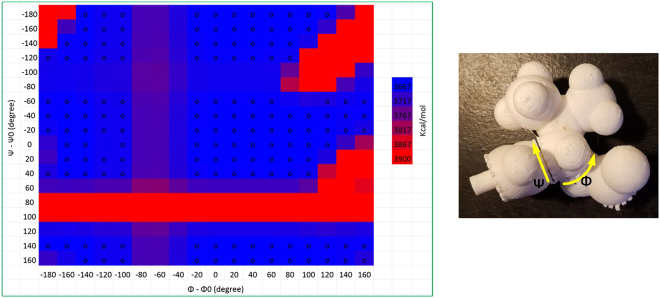
Three-component physical model is tested at 18 × 18 combinations of *ϕ* and *ψ* to investigate which ones are physically plausible. Further a heat map of energy is generated using the software.

One of the inherent features of the Assemble-And-Match tool is the capability of avoiding molecular conformations and assemblies that are highly unfavorable (i.e., those found at very high free energy levels) by simply taking advantage of physical collision. To investigate this property, a Ramachandran plot^48^ was generated using the physical model and the developed software (which has the Amber force field embedded) to see how well the two match. The molecular fragments used for generating the plot were extracted from a protein chain with the PDB code 2MZU. The main chain dihedral angles *ϕ* and *ψ* of the second amino acid (Val) were altered by increments of 20 degrees to form an 18 × 18 mesh. Figure 5 overlaps the heat map of free energy obtained from the software and the physically reachable dihedral angle combinations (denoted with **o** marks) obtained manually. A great match is observed even though atom van der Waals radii are scaled 0.7 times to expand the conformation space of the model (Note that there is a trade off between adopting atom radii near their van der Waals values and the size of the conformation space. Our experience shows a scale down of 0.7 yields a pretty handy model. Our selection is also in agreement with the claim made in ref.^16^). Note that the axes of the plot reflect deviations from the original *ϕ* and *ψ* values (extracted from PDB) and not *ϕ* and *ψ* themselves.

### Quantitative Evaluation of the Geometric Match Quality

**Figure 6:**
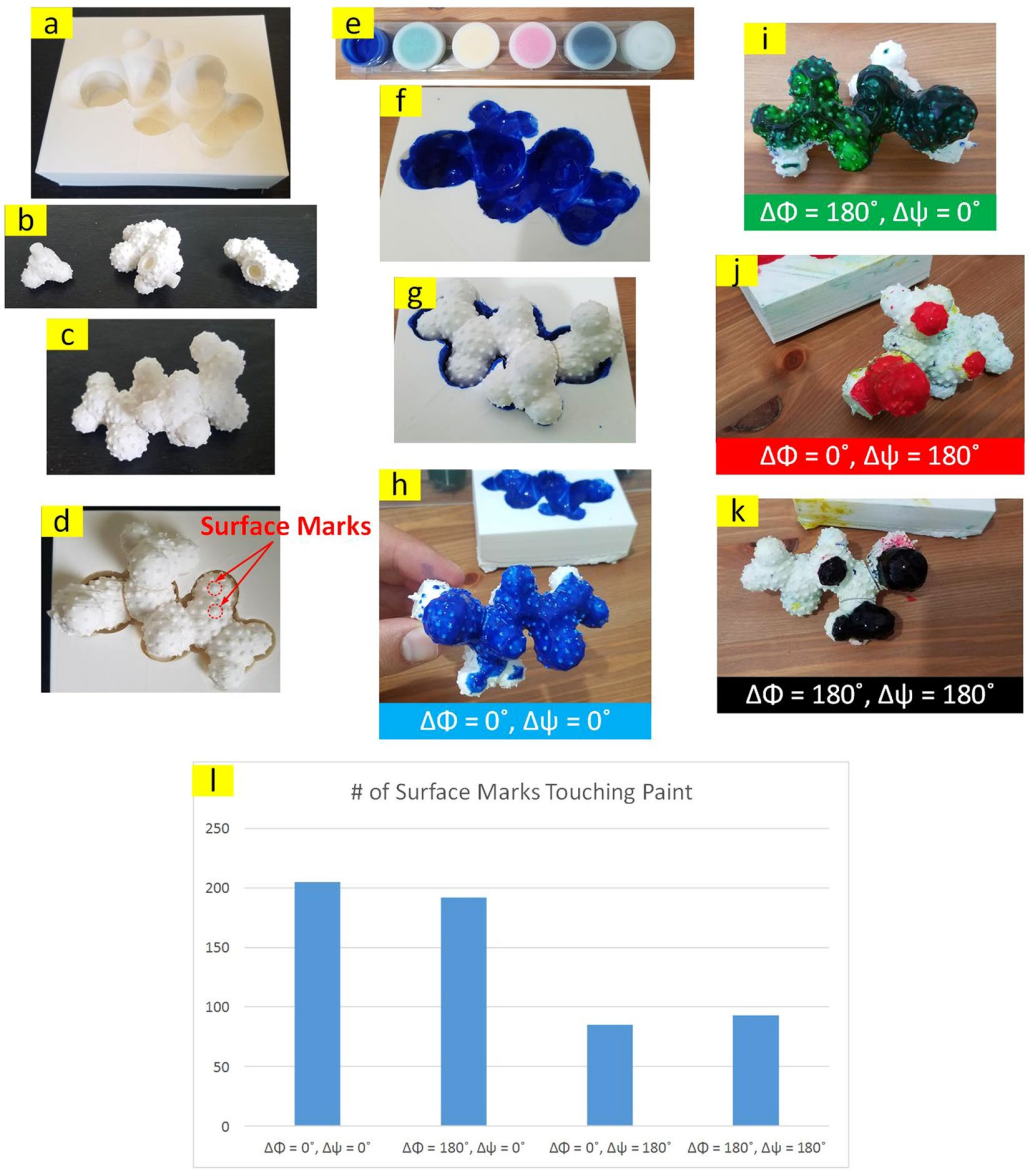
(**a**) 3D printed template of the ligand acting as the receptor. (**b**) 3D printed ligand fragments with surface marks. (**c**) Ligand fragments assembled at native dihedral angles extracted from PDB. (**d**) Geometric match of ligand and receptor. (**e**) Paint. (**f**) Painted receptor pocket. (**g, h**) Ligand touching the painted receptor at native dihedral angles, i.e. ∆*ϕ* = 0° and ∆*ψ* = 0°. (**i**) Ligand touching the painted receptor at ∆*ϕ* = 180° and ∆*ψ* = 0°. (**j**) Ligand touching the painted receptor at ∆*ϕ* = 0° and ∆*ψ* = 180°. (**k**) Ligand touching the painted receptor at ∆*ϕ = *180° and ∆*ψ* = 180°. (**l**) Number of surface marks touching paint at 4 different conformations.

Finding the best (geometric) match between the ligand and receptor is key in structure based drug design. Thus, it is critical for a physical model to be capable of reflecting the match quality in a comparative fashion to differentiate between promising and poor molecular pairs. To benchmark our tool against this criterion, we extracted the starting section of a protein with PDB code 1L2Y to form a 3-piece ligand. Approximately equally distanced surface marks were embedded on the spherical atoms as a means of representing surface area in a discrete fashion. We also made the semi-perfect template (as described above) as the receptor for this ligand (such that perfect match occurs between ligand and receptor at the PDB torsion angles). This was followed by measuring and comparing the contact area between the ligand and the receptor at different conformations (i.e., different selections of the two torsion angles). For that the binding site on the receptor was painted with a water based color and then each conformer of the ligand was placed inside the pocket. For the sake of having a reasonable comparison, the third fragment’s position and orientation were preserved as much as physically possible. Needless to say, the parts of the ligand that were in contact with the receptor’s pocket got painted. The surface area that was painted was measured by counting the number of surface marks that were colored (Fig. 6). It is observed from Fig. 6l that the native conformation (i.e., ∆*ϕ* = 0° and ∆*ψ* = 0°) has resulted in the largest contact area which is in agreement with our expectations.

### An Existing Ligand/Receptor Example

**Figure 7:**
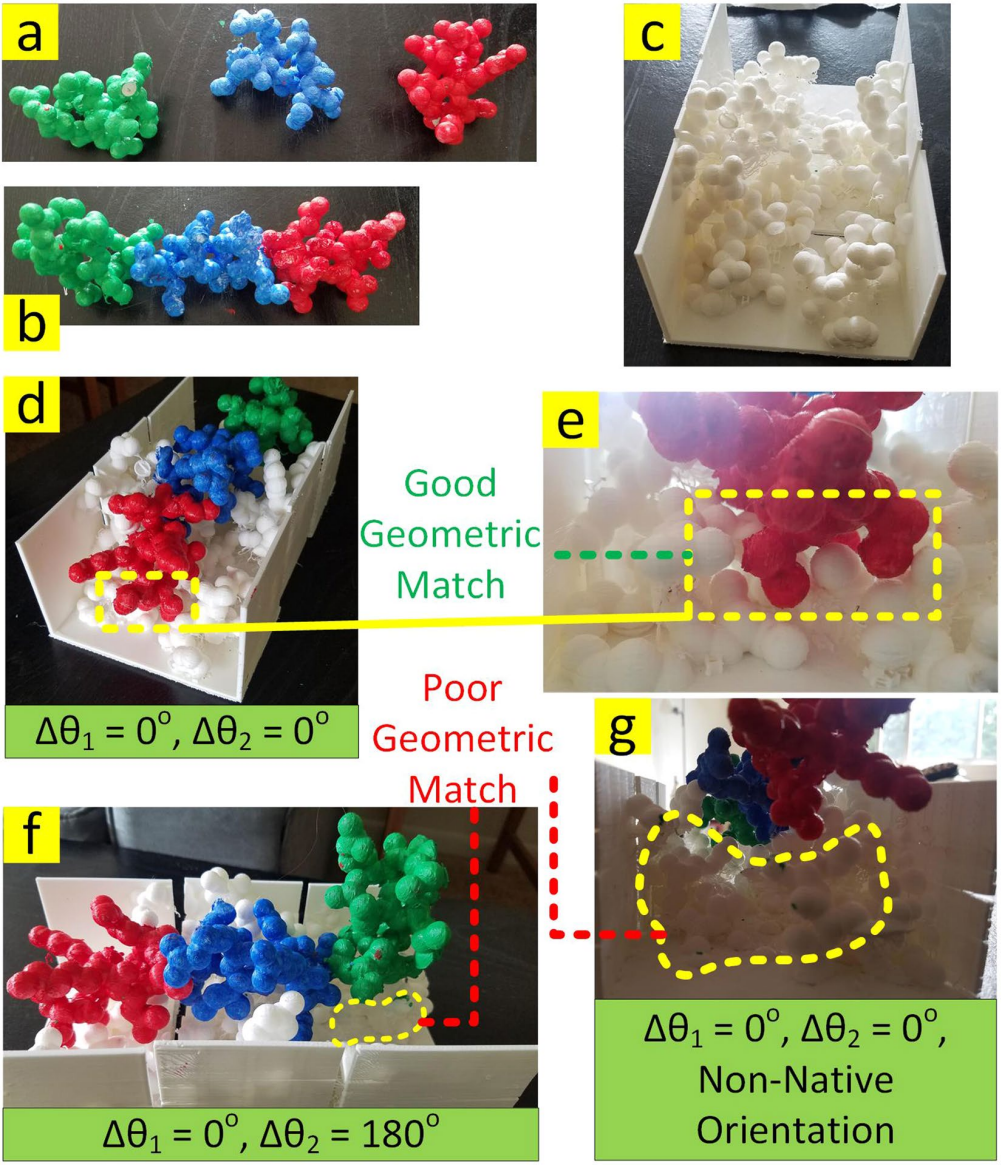
Reproducing the ligand/receptor complex found in PDB 3PL7. (**a**) Coarse fragmentation of the ligand. (**b**) Ligand chain with connected pieces. (**c**) Binding site of the receptor segmented into 3 pieces to make 3D printing possible on a small platform. (**d**) Ligand/receptor at native torsion angles and orientation. (**e**) Magnified view of **d**. (**f**) Non-native setting of one of the torsion angles. (**g**) Wrong orientation of ligand relative to the receptor.

An existing ligand/receptor example, with the PDB code of 3PL7, was used as a benchmark for Assemble-And-Match tool to examine its capability of effectively representing the real world molecular complexes. The ligand was coarsely fragmented into 3 pieces where each piece contained about 7 amino acids (Fig. 7a,b). Also, the binding site on the receptor was located and then segmented into 3 thirds to make the 3D printing possible with the small 3D printing platform available for this project (Fig. 7c). Despite the fact that the hydrogen atoms were not included in the model, the atom radii were scaled 0.7 times and some printer errors were present for this example, it is observed that for proper selection of the two torsion angles (between pieces 1 and 2 as well as pieces 2 and 3), and correct orientation of ligand and receptor, a reasonable geometric match will be attained (Fig. 7d,e). This is while non-native setting of even one torsion angle (Fig. 7f) or choosing the wrong orientation between ligand and receptor (Fig. 7g) leads to poor geometric matching.

### Size Customization

**Figure 8:**
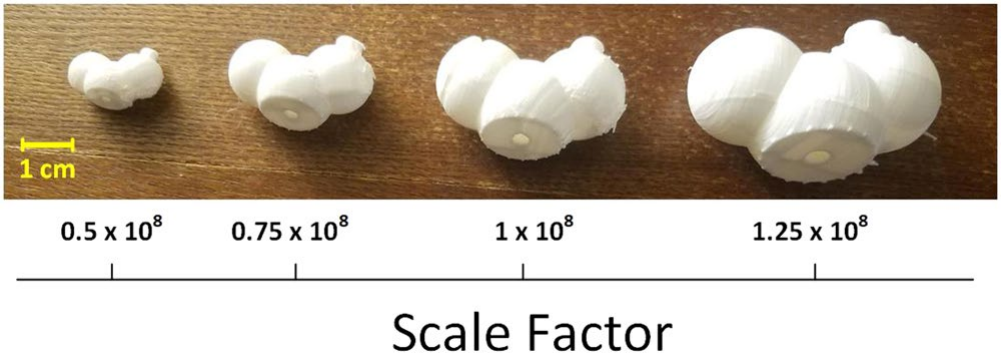
Scale factor is adjustable by the user based on their needs. Here, the same ligand fragment is printed with 4 different scale factors. The value of the scale factor reflects the ratio of a distance in the physical molecular model to the corresponding distance in the real molecule.

Depending on the size of the receptor molecule and the number of fragments that construct the ligand, the option of adjusting the scale factor is provided to the user. Figure 8 demonstrates the same ligand fragment printed in 4 different scales. As a result, unlike the prebuilt models (e.g., commercial ball and stick models), in which, hand-manipulation of the model becomes substantially difficult when molecular size increases over a certain extent, using Assemble-And-Match, even very large molecules can still be handled conveniently.

### Mobility of Receptor’s Side Chains

**Figure 9:**
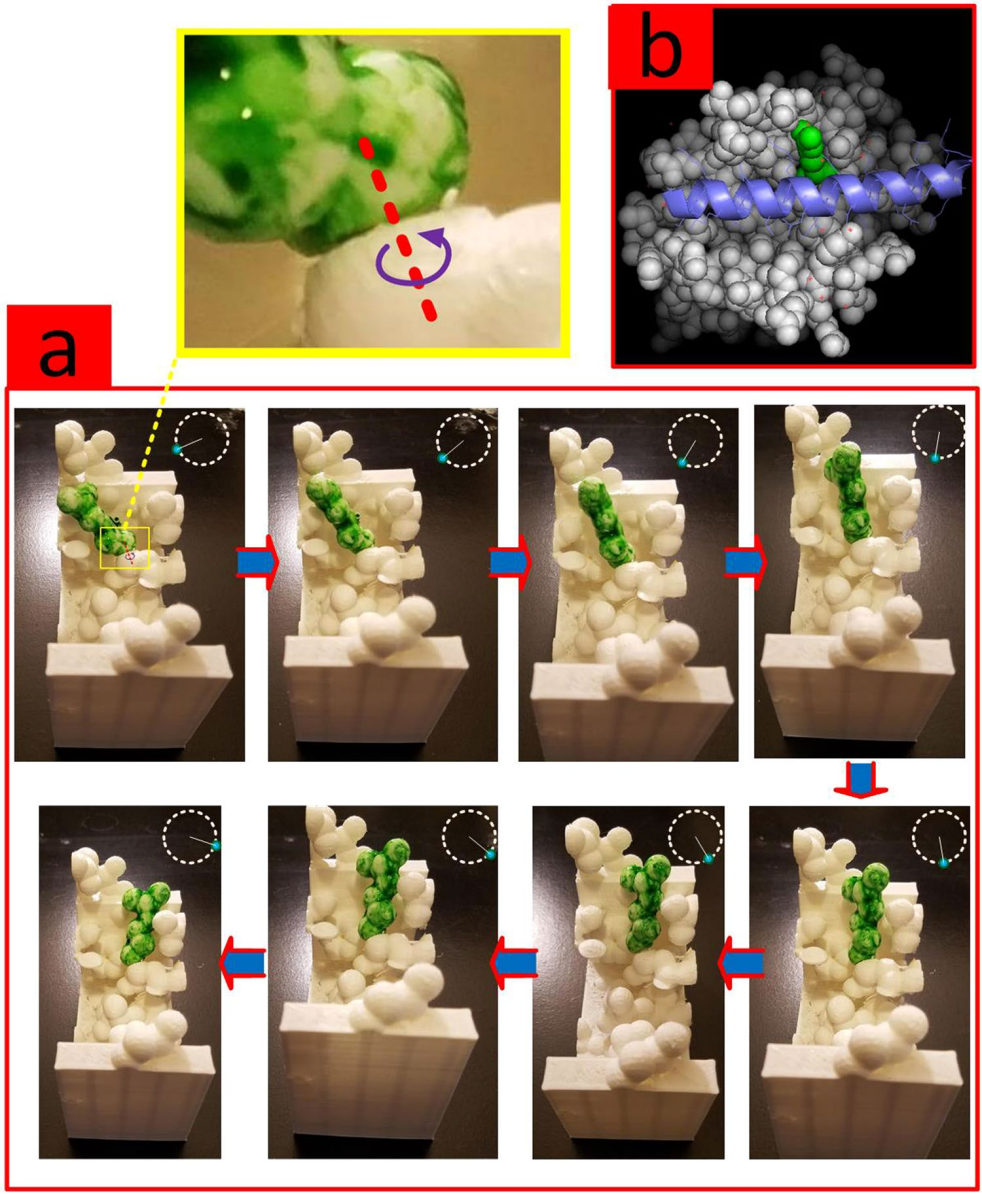
(**a**) Side chain mobility of amino acid R139 (in green) of the target through rotation about the covalent bond between *α* and *β* carbons is shown. (**b**) The ligand/receptor complex in PyMOL^51^. Receptor, R139 and ligand are shown in white, green and blue respectively.

Enabled by the software, the side chains of the target molecule can be optionally mobile. Figure 9 illustrates the mobility of a side chain from the target molecule in the ligand/receptor complex with PDB code 3PL7.

### Charged-Charged Interactions

**Figure 10:**
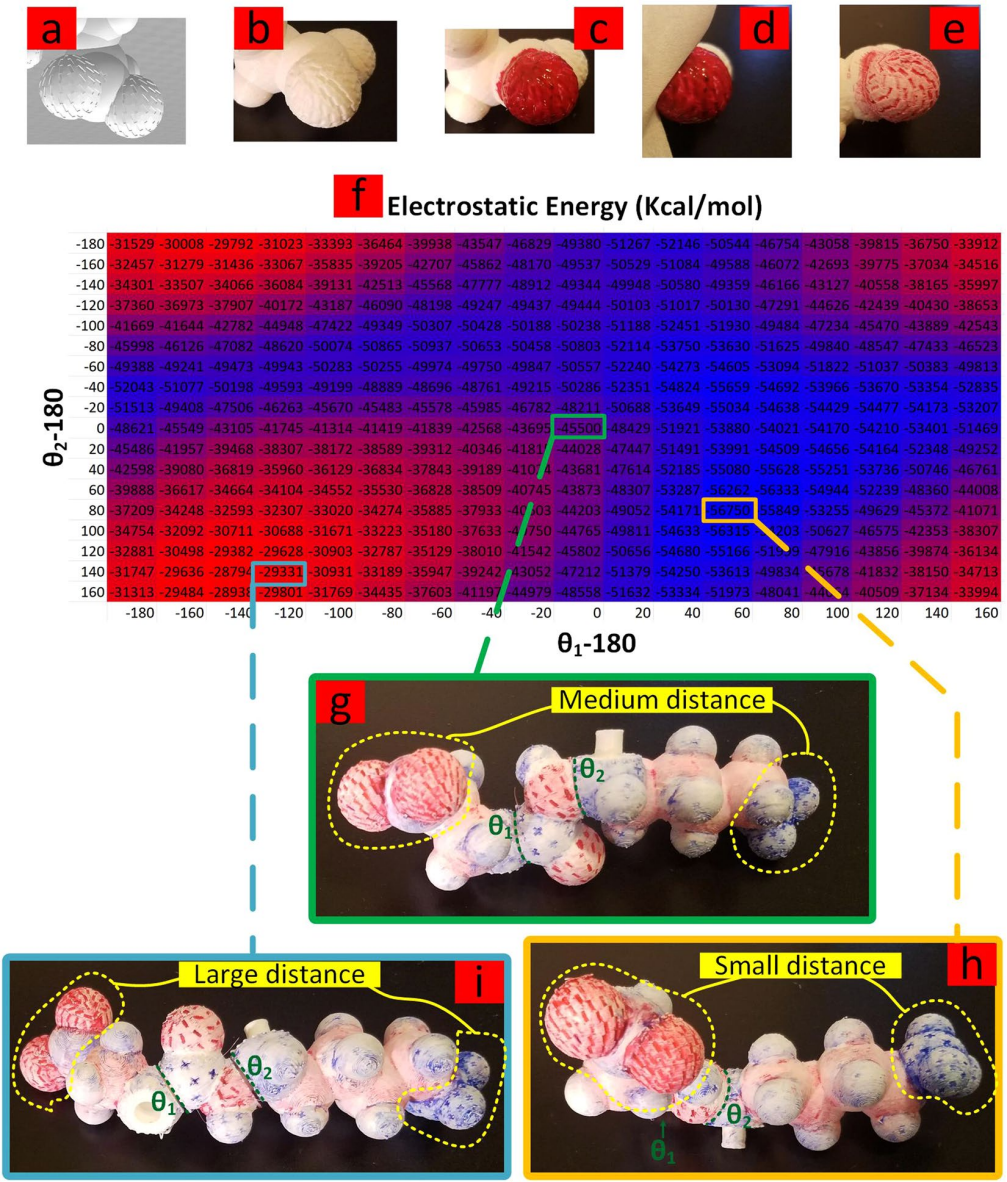
(**a**) Automatically embedded charge marks on the atoms’ surfaces in the CAD model. (**b**) Charged marks in the 3D printed model. (**c**) Paint used to color a partially negatively charged atom. (**d**) Wiping paint from the surfaces. (**e**) Charged marks remained colored after wiping the paint. (**f**) The heat map of electrostatic energy generated for a 3–piece chain consisting of one negatively charged side chain (GLU) and one positively charged side chain (LYS), at *pH* = 7, flexible via two main chain angles of *θ*_1_ and *θ*_2_; Different levels of electrostatic energy for relatively (**g**) medium, (**h**) small, and (**i**) large distances of the two regions in which the opposite charges are concentrated.

To provide a visual guide for electrostatic interactions to the user, when manipulating the physical model, the software can embed charge marks (+ or −) on the spheres that represent atoms, in a way that the population of the charge marks reflects the partial charge of the corresponding atom. After printing and following a simple painting/wiping step performed by the user, using red and blue colors, representing negative and positive charges respectively (this step can be eliminated if multi-material 3D printing is employed), the physical model now helps the user get a qualitative sense of favorable and unfavorable electrostatic energies. Figure 10, shows a 3–piece chain consisting of one negatively charged side chain (GLU) and one positively charged side chain (LYS), at *pH* = 7, flexible via two main chain angles of *θ*_1_ and *θ*_2_. The heat map of electrostatic energy has been generated for an 18 × 18 mesh of the two angles. The correlation between the distance of the two regions in which the opposite charges are concentrated, namely the protonated/deprotonated parts of the two side chains (indicated by yellow dotted lines in Fig. 10), and its effect on the overall electrostatic energy can be observed through the 3 conformations shown in Fig. 10. It is worth emphasizing however, that, such qualitative measure is integrated by accurate computations provided by the software at any reported conformation to yield the best results. As a manifestation of the easy connection with other software, the molecular coordinates of this example were first generated via Avogadro software^49^, and then fed to Assemble-And-Match software using mol2 data file type.

Also, enabled by the software, the printed models can visually guide the user towards proper formation of hydrogen bonds, in terms of distance and direction.

## Limitations and Future Directions

Despite several automated features of the tool, there are certain cases in which users’ manual action is still required, some of which are subject to automation in the following versions. These include but are not limited to reporting (in the software) the peptide sequence and the torsion angles as well as the spatial pose of the final conformation of the ligand with respect to the receptor to establish instantaneous communication between physical and *in silico* models. Special mechanisms and visual tools are currently under development to address these areas. Moreover, we are working on a novel analog computing method for evaluating the quality of matches which can make a breakthrough in the area of shape complementarity, in general and structure-based drug design, in particular.

**Figure 11:**
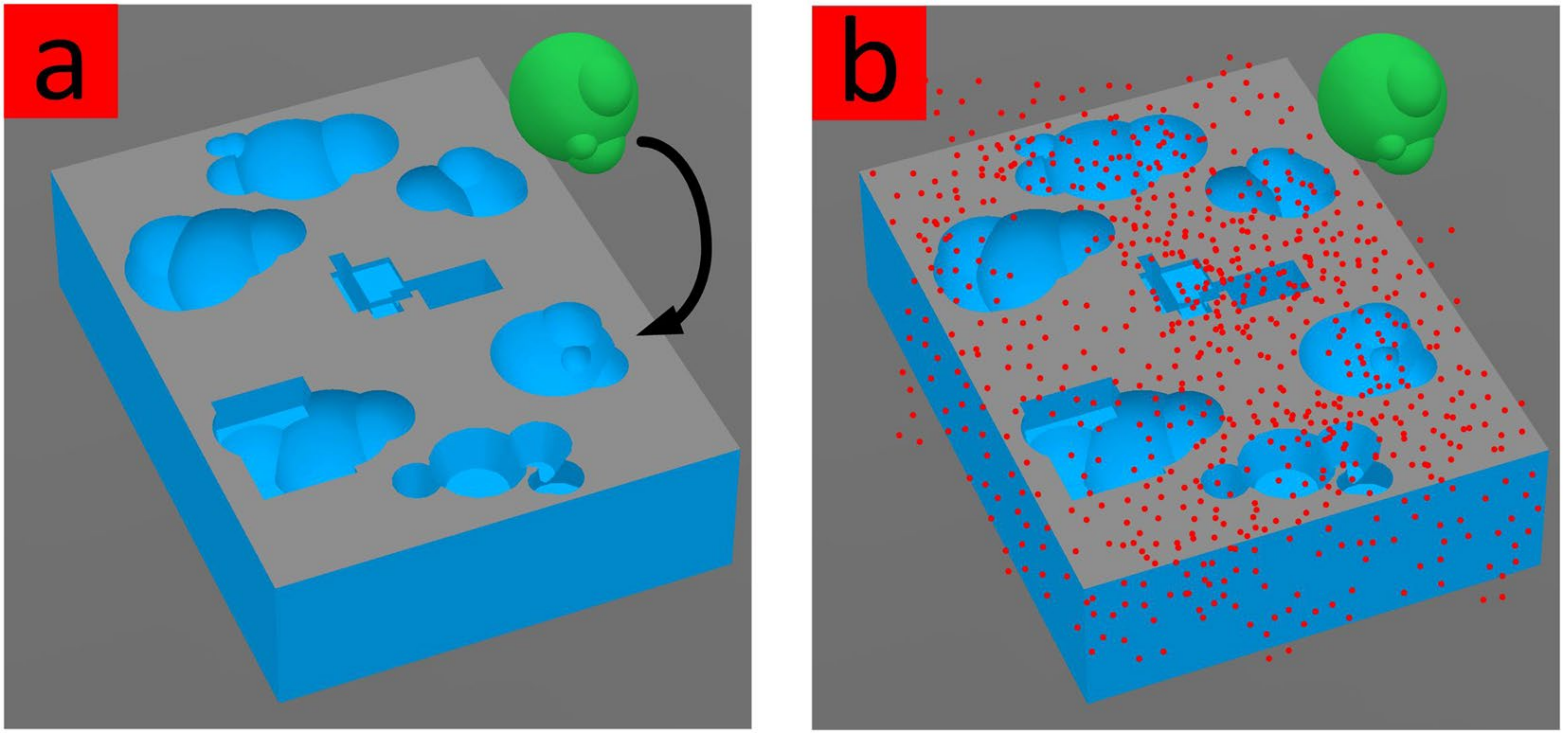
(**a**) Instantaneous recognition of the potential match by the cognitive mind. (**b**) Computer screening through extensive sampling.

Assemble-And-Match serves as a powerful tool for students and instructors for better comprehension and teaching of biochemistry concepts and for researchers to enjoy a tangible interface while benefiting from accurate *in silico* computations. Although, the educational aspect might be manifested more strikingly in the current version, the prospective tool is expected to provide more efficient screening processes by exploiting the cognitive abilities of humans. Inspired by Foldit^50^, a crowd-sourcing computer game which enables its players to contribute to solving the protein folding problem, we envision that Assemble-And-Match can become a crowd-sourcing physical game for drug discovery purposes. Perhaps, the exaggerated example illustrated in Fig. 11 can show how the idea of combining human cognition with computer power can be seeded towards establishing more effective drug design methods in the future.

## Conclusion

We reported the design and validation of a hybrid tool for rational structure-based drug design, Assemble-And-Match, consisting of computational and physical modules. The fact that the physical model is highly tangible and interactive and yet can be measured and evaluated using software has empowered the combination of human sensory and intuition with the *in silico* computational power for solving the drug design problem. The developed tool is highly detailed and customizable. More so, its plug-and-play feature supports rapid investigation of various molecular combinations, which in fact is ideal for structure based drug design applications. The conducted tests on the first version of our design promise a tool that can be used, as is, by the experts in the field of drug design, for instance, by providing a more convenient way of communication between experimental and computational scientists through a model that is physically tangible and yet, offers accurate computations. Further, the tool has a great potential to be enhanced such that it can also be used by non-experts in a way that it promotes democratization of drug design.
